# Evolution and Ecophysiology of the Industrial Producer *Hypocrea jecorina* (Anamorph *Trichoderma reesei*) and a New Sympatric Agamospecies Related to It

**DOI:** 10.1371/journal.pone.0009191

**Published:** 2010-02-12

**Authors:** Irina S. Druzhinina, Monika Komoń-Zelazowska, Lea Atanasova, Verena Seidl, Christian P. Kubicek

**Affiliations:** Research Area of Gene Technology and Applied Biochemistry, Institute of Chemical Engineering, Vienna University of Technology, Vienna, Austria; Cinvestav, Mexico

## Abstract

**Background:**

*Trichoderma reesei*, a mitosporic green mould, was recognized during the WW II based on a single isolate from the Solomon Islands and since then used in industry for production of cellulases. It is believed to be an anamorph (asexual stage) of the common pantropical ascomycete *Hypocrea jecorina*.

**Methodology/Principal Findings:**

We combined molecular evolutionary analysis and multiple methods of phenotype profiling in order to reveal the genetic relationship of *T. reesei* to *H. jecorina*. The resulting data show that the isolates which were previously identified as *H. jecorina* by means of morphophysiology and ITS1 and 2 (rRNA gene cluster) barcode in fact comprise several species: i) *H. jecorina/T. reesei* sensu stricto which contains most of the teleomorphs (sexual stages) found on dead wood and the wild-type strain of *T. reesei* QM 6a; ii) *T. parareesei* nom. prov., which contains all strains isolated as anamorphs from soil; iii) and two other hypothetical new species for which only one or two isolates are available. *In silico* tests for recombination and *in vitro* mating experiments revealed a history of sexual reproduction for *H. jecorina* and confirmed clonality for *T. parareesei* nom. prov. Isolates of both species were consistently found worldwide in pantropical climatic zone. Ecophysiological comparison of *H. jecorina* and *T. parareesei* nom. prov. revealed striking differences in carbon source utilization, conidiation intensity, photosensitivity and mycoparasitism, thus suggesting adaptation to different ecological niches with the high opportunistic potential for *T. parareesei* nom. prov.

**Conclusions:**

Our data prove that *T. reesei* belongs to a holomorph *H. jecorina* and displays a history of worldwide gene flow. We also show that its nearest genetic neighbour - *T. parareesei* nom. prov., is a cryptic phylogenetic agamospecies which inhabits the same biogeographic zone. These two species thus provide a so far rare example of sympatric speciation within saprotrophic fungi, with divergent ecophysiological adaptations and reproductive strategies.

## Introduction

The holomorphic fungal genus *Hypocrea/Trichoderma* (Hypocreales, Ascomycota) contains several hundred species comprising both sexually propagating (*Hypocrea*) as well as apparently mitosporic (*Trichoderma*) taxa without observed ability to sexual reproduction. One species of *Trichoderma* -*T. reesei* - is particularly well known because it is used in the biotechnological industry for the production of cellulolytic and hemicellulolytic enzymes and recombinant proteins [1], [2]. This property, and strategies for its further improvement, have recently regained strong interest because of the attempts to produce second generation biofuels to combat carbon dioxide emission and dependence on fossil oil [3], [4].

*T. reesei* was originally isolated as in the Solomon Islands during WW II, where it destroyed canvas and other cellulose-containing material of the US army [5]. This species is unique among industrial fungi, as it was known only from this single wild-type isolate ( = QM 6a) for last 50 years, and all mutant strains used in biotechnology today have thus been derived from it. Kuhls et al. [6], however, used molecular characters (internal transcribed spacers 1 sequences of the rRNA gene cluster and RAPD fingerprinting) to conclude that *T. reesei* was indistinguishable from the pantropical ascomycete *Hypocrea jecorina,* and suggested that the latter species is its teleomorph. Yet, although *H. jecorina* is heterothallic [7], the authors were unable to mate *T. reesei* with wild type isolates of *H. jecorina in vitro*. Further, Kuhls et al. [6] noted small phenotypic differences between *T. reesei* QM 6a and wild-type strains of *H. jecorina*. Consequently, the authors suggested that *T. reesei* is a clonal derivate from *H. jecorina* but this argument was not considered in the taxonomic status of the first fungus. Seidl *et al*. [8] recently described that *T. reesei* can indeed be crossed with wild-type isolates of *H. jecorina*, thus rejecting at least one of the above arguments for the clonality of *T. reesei*.

The progress in molecular evolutionary tools and theoretical concepts for species recognition has recently led to the detection of a continuously increasing number of cryptic species within individual morphological or biological fungal taxa [9]–[13] including *Hypocrea/Trichoderma* [14]–[16]. Recent world-wide sampling has detected a putative anamorph of *H. jecorina* as being common in soils of South East Asia and particularly in South America [17]–[19]. The frequent occurence of these strains in soil led us to hypothesize that they may eventually be co-specific with *T. reesei*, and the latter in fact is not being an anamorph of but a cryptic species to *H. jecorina*. The attribution of *T. reesei* to holomorph of *H. jecorina* has so far been only claimed from identical ITS 1 and 2 sequences, but recent studies have shown that sequences from this locus are unable to distinguish between several closely related *Hypocrea/Trichoderma* species [15]–[17].

Here we will show that *T. reesei* sensu stricto ( = QM 6a) indeed belongs to the holomorphic species *H. jecorina*, and exhibits a history of recombination and world-wide gene flow, thus rejecting the hypothesis of being a genetically separated agamospecies derived from *H. jecorina*. In addition, we will also show that strains recently isolated from soils as putative anamorphs of *H. jecorina* in fact form two sibling species and that at least one of them (*T.* parareesei nom. prov.) reproduces asexually, and is the result of sympatric speciation in parallel to *H. jecorina*. Ecophysiological analyses reveal that this sympatric speciation is due to the adaptation to two different habitats.

## Results

### Sample Design and Genetic Markers

Our sample consisted of 34 strains from teleomorphs and anamorphs (Table 1, already sorted in relation to the results from phylogenetic analysis given below), which were originally identified as *H. jecorina* or *T. reesei* both by morphological analysis as well as ITS1 and 2 based oligonucleotide barcode (*TrichO*Key [17]; online at www.ISTH.info). They covered the whole geographic variability known for these species (South and Central America, Caribbean archipelago, Africa, South Pacific, South East Asia, Macro-and Micronesia and the Indian subcontinent).

**Table 1:** Strains of *H. jecorina* sensu lato used in this study.

Taxon	C.P.K. strain Nr	Other strain Nrs	Substratum	Origin	Mating type (*MAT* locus)	GeneBank Nr			
						*MAT1-1-2/MAT1-1-3 or MAT1-2*	*tef1*	*cal1*	*las1*
*Hypocrea jecorina*	**160**	G.J.S. 85-236, [6]	arecoid palm	Indonesia, North Sulawesi	*1*–*2*		GQ354342	GQ354276	GQ354308
	**1285**	G.J.S. 86-408, [6]	unknown	Brazil, Para	*1*–*1*		n/a	GQ354295	GQ354329
	**1286**	G.J.S. 88–6, [6]	unknown	Brazil, Para	*1*–*1*		GQ354362	GQ354296	GQ354330
	**1274**	C.T.R. 72–94, [6]	wood	Venezuela	*1*–*1*		GQ354357	GQ354292	GQ354325
	**170**	G.J.S. 86–410, [6]	bark	French Guiana	*1*–*1*		GQ354346	GQ354280	GQ354312
	**1273**	A.Y.R. 2896, [6]	dead log	French Guiana	*1*–*2*		GQ354356	GQ354291	GQ354324
	**1283**	G.J.S. 86–403, [6]	bark of recently dead tree	French Guiana	*1*–*1*		GQ354360	GQ354294	GQ354328
	**282**	G.J.S. 97–177, CBS 102271 [38]	fallen twig of *Theobroma*	French Guiana	*1*–*1*	GQ167152/GQ167152	GQ354347	GQ354281	GQ354313
	**283**	G.J.S. 97–178, CBS 102270 [38]	fallen twig of *Theobroma*	French Guiana	*1*–*1*		GQ354348	GQ354282	GQ354314
	**1392**	G.J.S. 86–401	unknown	Puerto Rico	*1*–*2*		GQ354368	GQ354302	GQ354336
	**1380**	G.J.S. 95–82, CBS 498.97	decorticated wood	Puerto Rico	n/a		GQ354364	GQ354298	GQ354332
	**1386**	G.J.S. 95–2081	bark	Puerto Rico	*1*–*1*		GQ354365	GQ354299	GQ354333
	**1387**	G.J.S. 95–2082	bark	Puerto Rico	*1*–*1*		GQ354366	GQ354300	GQ354334
	**1388**	G.J.S. 95–123	unknown	Puerto Rico	*1*–*1*		GQ354367	GQ354301	GQ354335
	**1282**	G.J.S. 85–249, [6]	log	Indonesia, North Sulawesi	*1*–*1*		GQ354359	GQ354293	GQ354327
	**158**	G.J.S. 85–229, [6]	unknown	Indonesia, North Sulawesi	*1*–*2*		GQ354343	GQ354277	GQ354309
	**159**	G.J.S. 85–230, [6]	wood	Indonesia, North Sulawesi	*1*–*1*		GQ354344	GQ354278	GQ354310
	**1407**	CBS 881.96	unknown	Papua New Guinea	*1*–*1*		GQ354369	GQ354303	GQ354337
	**1127**	G.J.S. 93–23, 6]	bark	New Caledonia	*1*–*2*		GQ354355	GQ354290	GQ354323
	**1337**	G.J.S. 93–22, [6]	decorticated wood	New Caledonia	*1*–*2*		GQ354363	GQ354297	GQ354331
	917	CBS 383.78, QM 6a, [6]	cellulose fabrics	Solomon Islands	*1*–*2*	GQ167145	Z23012	n/a	GQ354321
	**3418**	G.J.S. 06–138	unknown	Cameroon	n/a		GQ354370	GQ354304	GQ354338
	**3419**	G.J.S. 06–140	unknown	Cameroon	n/a		GQ354371	GQ354305	GQ354339
	**938**	G.J.S. 89–7, CBS 836.91, [6]	bark	Brazil	*1*–*2*		GQ354354	GQ354289	GQ354322
	**155**	G.J.S. 86–404, [6]	unknown	Brazil, Para	*1–1*		GQ354345	GQ354279	GQ354311
*H.*sp. G.J.S. 85–238	**1281**	G.J.S. 85–238, [6]	wood	Indonesia, North Sulawesi	n/a		GQ354358	n/a	GQ354326
*Trichoderma parareesei* nom. prov.	3420	G.J.S. 04–41	soil	Brazil	n/a		GQ354372	GQ354306	GQ354340
	3426	G.J.S. 07–26	soil	Ghana	n/a		GQ354373	GQ354307	GQ354341
	661, [17]		soil	Argentina	*1–2*			GQ354286	GQ354318
	665, [17]		soil	Argentina	*1–1*	GQ167143/GQ167148	GQ354352	GQ354287	GQ354319
	717, [17]		soil	Mexico	*1*–*1*	GQ167144/GQ167149	GQ354353	GQ354288	GQ354320
	634, [17]		soil	Sri Lanka	*1–1*	GQ167142/GQ167147	GQ354351	GQ354285	GQ354317
*T.* sp. nov. C.P.K. 524	523, [19]		rotting wood	Taiwan	*1–1*		GQ354349	GQ354283	GQ354315
	524, [19]		rotting wood	Taiwan	*1–1*	GQ167141/GQ167146	GQ354350	GQ354284	GQ354316

bold font indicates strains isolated from fruiting bodies (teleomorphs), n/a corresponds to not available

**Figure 1:**
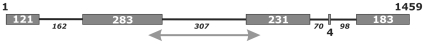
Structure of the *las1* locus in *H. jecorina/T. reesei*. Intron-exon structure of the *las1* locus in *H. jecorina/T. reesei* and position of PCR primers as inferred for *T. reesei* QM 6a.

A preliminary screening for phylogenetic markers, which were previously used with success in other studies on *Hypocrea/Trichoderma* (such as *tef1, cal1, chi18-5, rpb2* or *act1* [15]–[17], [20]), showed that only the 4th intron of *tef1* and the 2^nd^ and 3^rd^ introns of *cal1* provided sufficient phylogenetic information. Coding regions such as *chi18-5* or *act1* provided insufficient polymorphism (data not shown). We therefore searched for additional genes with long introns and tested their ability to differentiate within *H. jecorina*. One locus fulfilling this requirement is the *las1* gene (fgenesh5_pg.C_scaffold_1000016), which encodes the orthologue of an essential nuclear protein regulating bud formation and morphogenesis in *Saccharomyces cerevisiae* [21]. It is interrupted by four introns, of which the second (307 nts) was selected as the phylogenetic marker (Fig. 1).

Sequencing of *tef1, cal1* and *las1* for the whole strain sample provided 1241 nts, of which 1098 were constant sites and 109 polymorphic characters were parsimony-informative (49 in *tef1*, 38 in *las1* and 22 in *cal1*, respectively). Nucleotide characteristics of the three genes are shown in Table 2.

**Table 2:** Nucleotide properties of phylogenetic markers and MCMC parameters.

Parameters		phylogenetic marker			
		*tef1*	*las1*	*cal1*	concatenated dataset
Fragment characterization		intron	exon/intron	exon/intron	not applicable
Number of sequences		33	33	33	33
Number of characters		509	377	355	1241
parsimony informative		18	9	7	34
constant		442	330	326	1098
**Parameters of MCMC analysis**					
Mean nt frequencies* A/C/G/T		0.21/0.32/0.22/0.25	0.20/0.24/0.34/0.22	0.24/0.28/0.29/0.19	0.22/0.28/0.27/0.23
Substitution rates*	A <-> C	0.02	0.09	0.08	0.09
	A <-> G	0.89	0.22	0.17	0.29
	A <-> T	0.01	0.19	0.16	0.1
	C <-> G	<0.01	0.08	0.06	0.04
	C <-> T	0.03	0.37	0.40	0.31
	G <-> T	0.04	0.06	0.13	0.17
alpha*		0.10	0.10	0.08	0.05
MCMC generations, million/number of runs		1/2	1/2	1/2	2/2
PSRF*		1.00	1.00	1.00	1
Number of chains/Temp (λ)		4/0.2	4/0.2	4/0.2	4/0.2
Sampling frequency		100	100	100	100
Number of discarded first generations		600	400	500	800
Total tree length		3.19	5.79	6.05	0.43

*as estimated after GTR MCMC sampling and burning.

### Molecular Phylogeny of *H. jecorina* Sensu Lato

**Figure 2:**
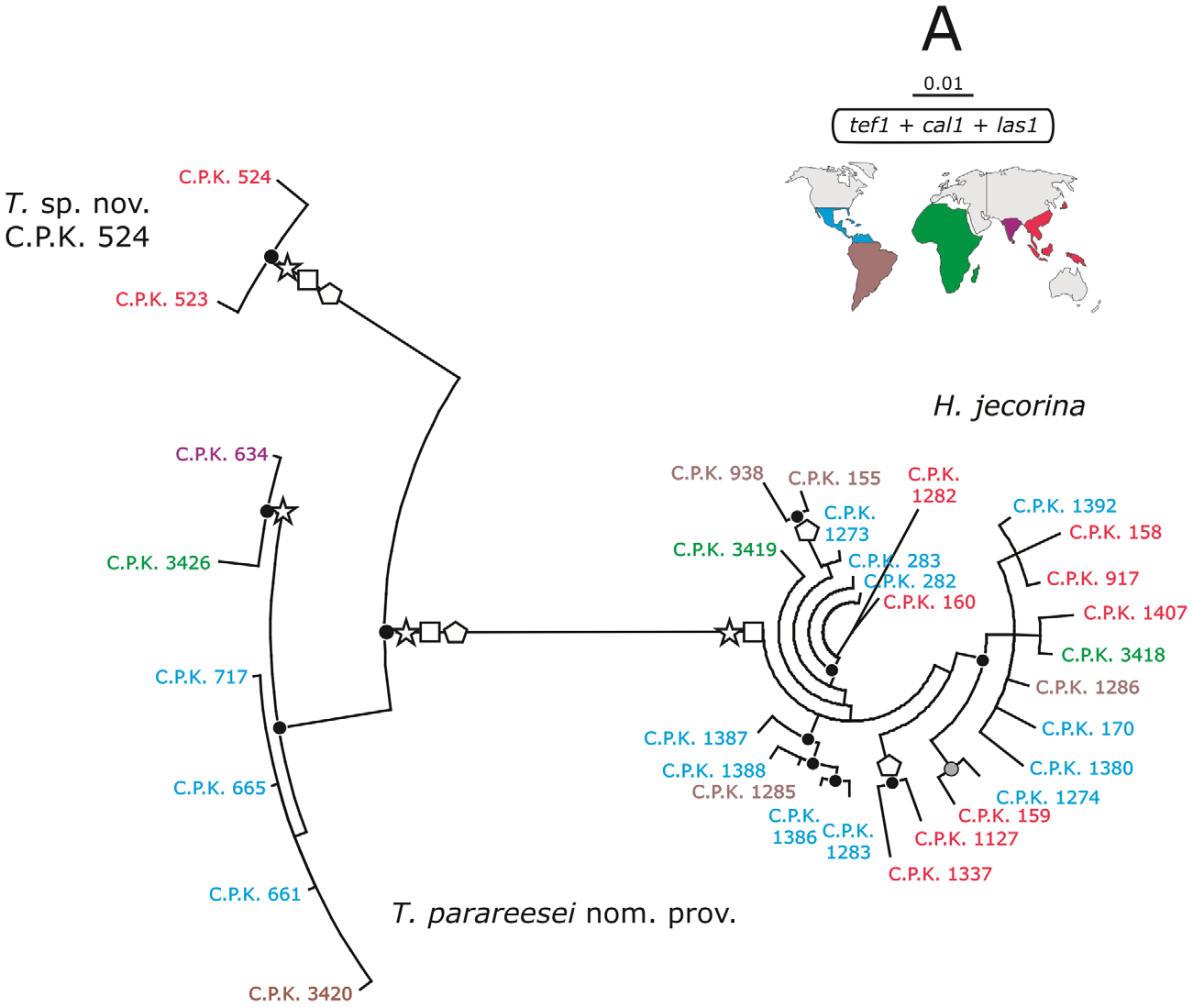
Molecular phypogeny of *H. jecorina* sensu lato. Bayesian circular phylogram inferred from the concatenated dataset of *tef1*, *cal1* and *las1* phylogenetic markers. Symbols at nodes correspond to posterior probabilities (PP) >95%. Filled circles correspond to PP in the concatenated tree, open stars, squares and polygons to PP in *las1*, *cal1* and *tef1* gene trees, respectively. The corresponding phylograms are given in Figure S1. The color code indicates the geographic region from which the isolates were obtained, as explained in the right top inset.

We used Bayesian phylogenetic analysis of both the concatenated as well as individual gene data sets to test for a phylogenetic structure within *H. jecorina* sensu lato (Fig. 2 and Fig. S1). One single teleomorph isolate from North Sulawesi, Indonesia (C.P.K. 1281 =  G.J.S. 85–238 - CBS 638.92), which was described as *H. jecorina* by Kuhls et al. [6] and Samuels et al. [22], consistently formed a single branch separated from all other clades in the *tef1*, *las1* and *cal1* trees (data not shown) indicating that it represents a still undescribed species outside the *H. jecorina* sensu lato clade. Thus, we named it as *H*. sp. nov. G.J.S. 85–238 and excluded it from further analyses. The remaining 33 isolates formed three significantly supported clades in all single gene trees as well as in the combined phylogram, thus fulfilling the criteria of the genealogical concordance phylogenetic species recognition concept [23], [24] which recognizes a clade as an evolutionary lineage if its separation is supported by at least two gene trees and not contradicted by the others. One of the obtained clades contained all the teleomorph isolates and also the original isolate of *T. reesei*. As the type strain of *H. jecorina* (specimen No. 989 of the Berkeley and Broom collection from Ceylon at Kew, UK) is not available for molecular analysis, we rely on the attribution of all these strains to the above taxon by Samuels et al. [22] and recognize this clade as a holomorph *H. jecorina/T. reesei* sensu stricto.

The next large clade contained all strains that were isolated from soil as anamorphs (Fig. 2). Since *T. reesei* was not clustered in this clade we call it *T*. sp. nov. ‘parareesei nom. prov.’ (for simplicity *T. parareesei* nom. prov.) throughout the manuscript, assuming that its formal taxonomic description will soon be published elsewhere. Two isolates (C.P.K. 523 and C.P.K. 524), collected from tree bark in Taiwan formed a separate branch in all of these trees and are therefore recognized as another phylogenetic species *T*. sp. nov. C.P.K. 524, whose formal description will be possible when additional isolates become available.

### Geographic Distribution of *H. jeco*rina and *T. parareesei* Nom. Prov

Our sample shows that both *H. jecorina* and *T. parareesei* nom. prov. are cosmopolitan species (Fig. 2), yet restricted to a narrow latitudinal belt around the equator (±20°). They must therefore be considered to be sympatric species, particularly in Central and South America. In order to test, whether the gene sequences would reveal some intraspecific geographic separation within *H. jecorina*, we determined the F_ST_ values for pairwise combinations of strains from different locations (e.g. Caribbean vs. Indopacific, South American vs. Africa etc.). However, the F_ST_ values were within 0.011–0.024 for all combinations (data not shown), thus documenting a high rate of exchange of genetic material over these wide geographic distances, and no evidence for geographic segregation.

### *In vitro* Mating between and within *H. jecorina* and *T. parareesei* Nom. Prov

**Figure 3:**
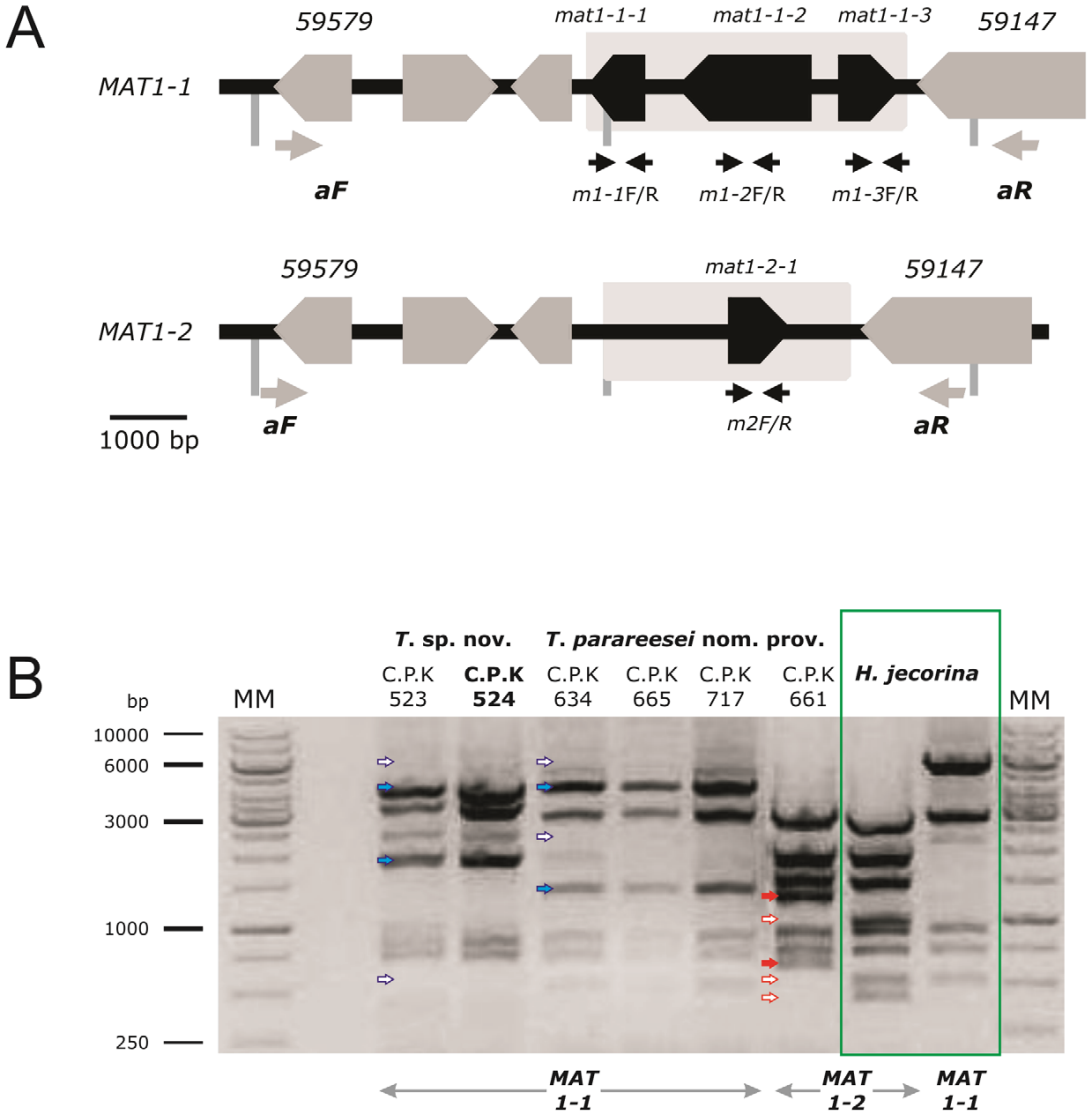
The mating type loci of *H. jecorina*. (A) Schematic presentation of the mating type loci *MAT1-1* and *MAT1-2* and their flanking regions based on the *H. jecorina* data [8]. Primers used to amplify the complete *MAT*-loci are indicated by gray arrows and primers for fragments of the mating type genes (Table 4) by black arrows. Numbers correspond to the respective proteins IDs in the *T. reeesei* genome database. (B) Restriction fragment patterns of the mating type loci, amplified with primers aF and aR (Table 4) and digested with *Pst*I. MM, molecular marker (GeneRuler 1 kb ladder, Fermentas). The strains and their respective mating types are indicated as C.P.K. numbers. Small colored arrows show either present (filled) or absent (open) bands in RFLP profiles of C.P.K. strains in respect to the reference strains of *H. jecorina* for *MAT1-1* and *MAT1-2* [8].

The mating type loci of the heterothallic species *H. jecorina* have recently been identified and conditions for successful mating have been established [8]. We thus tested whether the two newly recognized species would contain one of the two mating types of *H. jecorina*. Using primers within conserved regions of the genes *mat1-1-1*, *mat1-1-2*, *mat1-1-3* (indicative for a *MAT1-1* mating type locus, Fig. 3A) and *mat1-2-1* (indicative for a *MAT1-2* locus, Fig. 3A), we in fact identified that all isolates of *T. parareesei* nom. prov. have a *MAT1-1* locus except C.P.K. 661 from northern Argentina, which has a *MAT1-2* locus (Table 1). Both isolates of *T.* sp. C.P.K. 524 also possess a *MAT1-1* locus. RFLP of the complete mating type loci in comparison with *H. jecorina* confirmed the segregation of all three species in three and two different haplotypes of *MAT1-1* and *MAT1-2* respectively (Fig. 3B). Although most of the SNPs of the mating type loci of *T. parareesei* nom. prov. and *T.* sp. C.P.K. 524 were silent mutations, only the amino acid sequence of MAT1-1-2 of *T. parareesei* nom. prov. was identical to that from *H. jecorina*, whereas it was polymorphic in three positions in *T*. sp. C.P.K. 524. The amino acid sequence of the MAT1-1-3 protein of *T. parareesei* nom. prov. differed in two residues from that of *H. jecorina* including one case where the helix breaker P was exchanged by a polar S residue. The most significant difference was observed in MAT1-2-1, which contained a PS insertion (Fig. S2). Thus these data demonstrate that the mating type proteins from *T. parareesei* nom. prov. have undergone alterations which may have affected their functionality.

**Figure 4:**
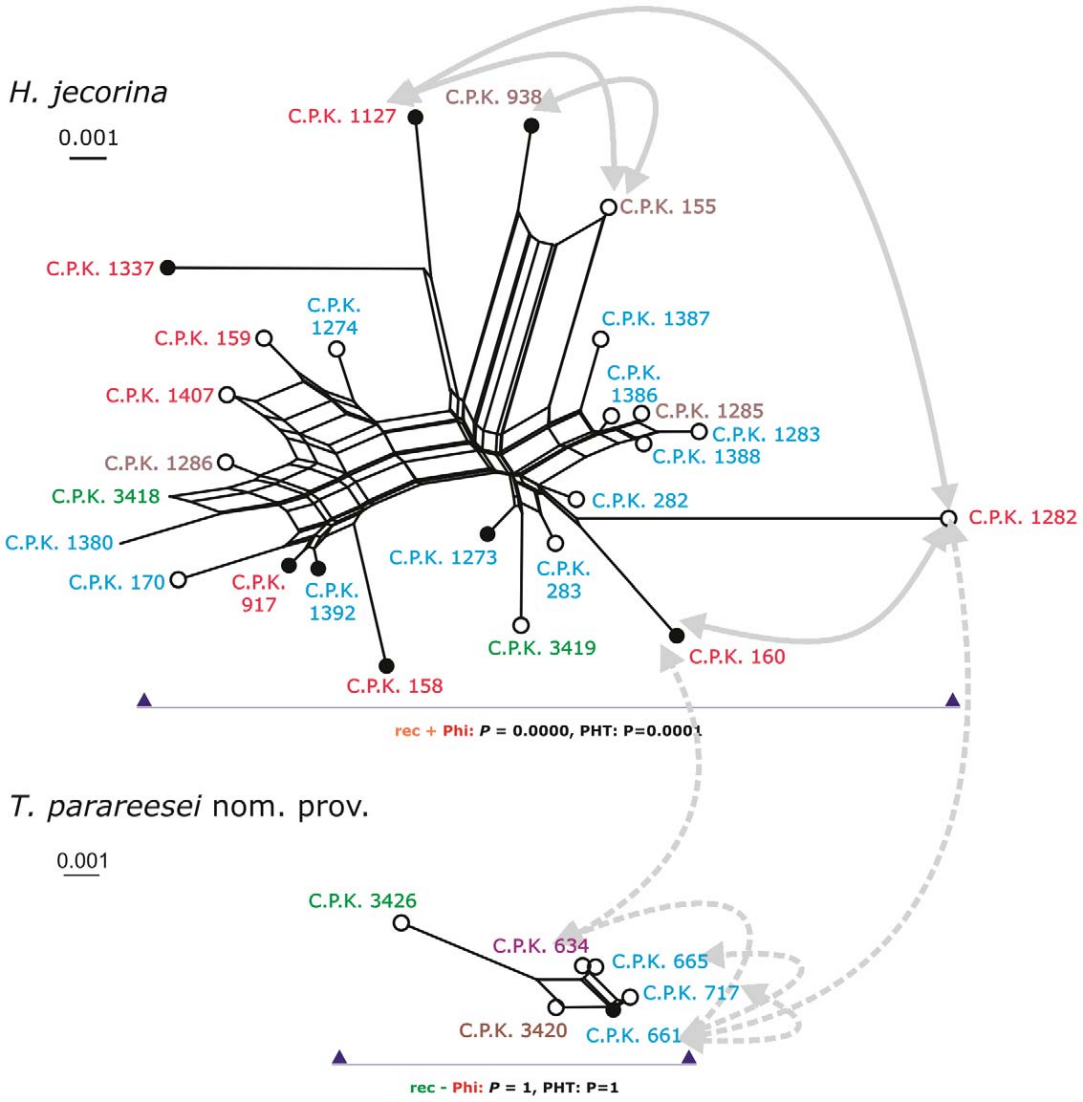
Recombination analysis of *H. jecorina* and *T. parareesei* nom. prov. Reconstruction of possible recombination networks build using the split decomposition method applied to the concatenated dataset (*tef1 + cal1 + chi18-5*). Upper shape: *H. jecorina*, low shape: *T. parareesei* nom. prov. Open and filled symbols at OTUs indicate *MAT1-1* and *MAT1-2* mating types respectively. Gaps were treated as missing characters throughout. All networks have been calibrated to fit one scale. The color scheme shows geographic origin of the strain as indicated in Fig. 2. Results from the PHT and Phi tests are shown by arrows and the respective P values, ‘rec +’ specifying positive recombination result and ‘rec -’specifying no recombination detected. PHT indicates the result of partition homogeneity test, Phi corresponds to results of Phi test. Double ended arrow lines show successful (solid line) and failed (dashed line) crossings.

The availability of two mating types for *T. parareesei* nom. prov. provided us with the possibility to test whether these isolates already developed an infertility barrier to *H. jecorina*, and if they would still be able to mate with each other. Therefore, selected isolates of *H. jecorina* and *T. parareesei* nom. prov. carrying the *MAT1-1* and *MAT1-2* locus respectively, were subjected to pairwise crossing experiments on plates under daylight conditions (indicated by arrows on Fig. 4). Only the heterothallic strain pairs belonging to *H. jecorina* indeed produced either mature fruiting bodies or primordia within 6–12 weeks of incubation, whereas all other combinations did not mate, even after further prolonged incubation time. These data are consistent with the conclusion that – at least in *vitro* - *T. parareesei* nom. prov. is unable to cross with *H. jecorina*, and is also not capable of sexual reproduction with the opposite mating partner of itself.

### *In silico* Analysis of Reproduction Strategies of *H. jecorina* and *T. parareesei* Nom. Prov

The results of mating experiments, the visual inspection of tree topologies (Fig. 2 and Fig. S1) and eventually the origin of strains (as teleomorph or anamorph, respectively) lead to the assumption that only *H. jecorina* performs sexual reproduction, whereas *T. parareesei* nom. prov. would be a clonal taxon ( = agamospecies). In order to investigate this by means of sequence analysis we first used the split decomposition method [25], [26] to test for the presence of network relationships in *H. jecorina* and *T. parareesei* nom. prov., using a concatenated dataset of *tef1, cal1* and *las1*. This method presents conflicting phylogenetic data, presumably arising from recombination, as an interconnected network of lineages. As shown in Fig. 4, such a network was evident within *H. jecorina* ( = the strains isolated as teleomorphs and *T. reesei*) whereas it was absent within *T. parareesei* nom. prov. We especially note that the *ex*-type strain of *T. reesei* was tightly linked to the *H. jecorina* network, thus arguing against its origin as a clonally separated isolate.

We then used the partition homogeneity test (PHT; [27], [28]) to examine the congruence between individual gene trees. This test produces artificial datasets by multiple (10 000) re-sampling and random swapping of observed datasets and subsequent construction of maximum-parsimony trees for every newly sampled ‘gene’ sequence. For clonally reproducing populations ( =  no sexual recombination), the sums of the lengths of the gene trees for the observed and re-sampled data should be similar. However, under recombination the sums of the tree lengths should be longer than those for the actual data because of introduction of homoplasy into unlinked sites. This test confirmed our analysis of topologies of single locus trees - the clades containing *T. parareesei* nom. prov. showed congruence of all three loci suggesting the clonality of this species (Fig. 4). The topologies of *H. jecorina* subclades appeared to be not concordant between individual trees, providing the evidence for recombination (Fig. 4).

As another means, we used the index of association (IA) test on a subset of ‘clone corrected’ data (i.e. individuals with identical alleles of the three loci were excluded so that each haplotype was represented only once; cf. [29]). In this test, complete panmixia (sexual compatibility resulting in recombination) would be indicated by a value of 0 ( = the null hypothesis). This value was neither obtained with the complete dataset nor with any of the individual clades (data not shown).

Consistent with the occurrence of teleomorphs *H. jecorina* clade gave a significantly lower value (0.014, *P* = 0.006), whereas strains of *T. parareesei* nom. prov. completely rejected the null hypothesis of recombination (1.060, *P* = 0.126).

Finally we applied the Phi-test, which uses the pairwise homoplasy index (PHI, Φ) to detect refined incompatibility [30]. This method assumes the infinite sites model of evolution in which the detection of incompatibility for a pair of sites indicates recombination. Application of this test to *H. jecorina* did find statistically significant evidence for recombination (P≪0.001) while it was not the case for *T. parareesei* nom. prov. (*P* = 1). Since the Phi-test is a very robust means which can detect recombination even in the presence of recurrent mutation, we decided to use this method to define the borders of recombining populations. To this end, we first detected a non-recombining subsample consisting of six most terminal strains of *H. jecorina* (C.P.K. 160 and 1282 from Indonesia, C.P.K. 155 and 938 from Brazil, C.P.K. 3419 from Cameroon and C.P.K. 1273 from Pacific; *P = 0.34*), and then gradually added strains from two other phylogenetic species until evidence for recombination was detected (*P*<0.05). No recombination was detected between *T. parareesei* nom. prov. and *H. jecorina*. In contrast, a positive recombination signal (*P* = 0.012) was obtained when both strains of *T.* sp. C.P.K. 524 were tested together with the above listed subset of *H. jecorina*. It suggests that *T. sp.* C.P.K. 524 may also reproduce sexually but the current sample is too small to reveal it.

Thus the four alternative tests suggest that *T. parareesei* nom. prov. is an agamospecies which did not undergo sexual recombination in its recent evolutionary history.

### Ecological Specialization of *H. jecorina* and *T. parareesei* Nom. Prov

The fact that *H. jecorina* and *T. parareesei* nom. prov. evolved in sympatry raises the question about the differences in their ecological niches. Since both fungi are saprotrophs, we tested whether they would differ in their carbon metabolism, response to environmental stimuli or antagonistic ability against other fungi.

#### Carbon utilization

**Figure 5:**
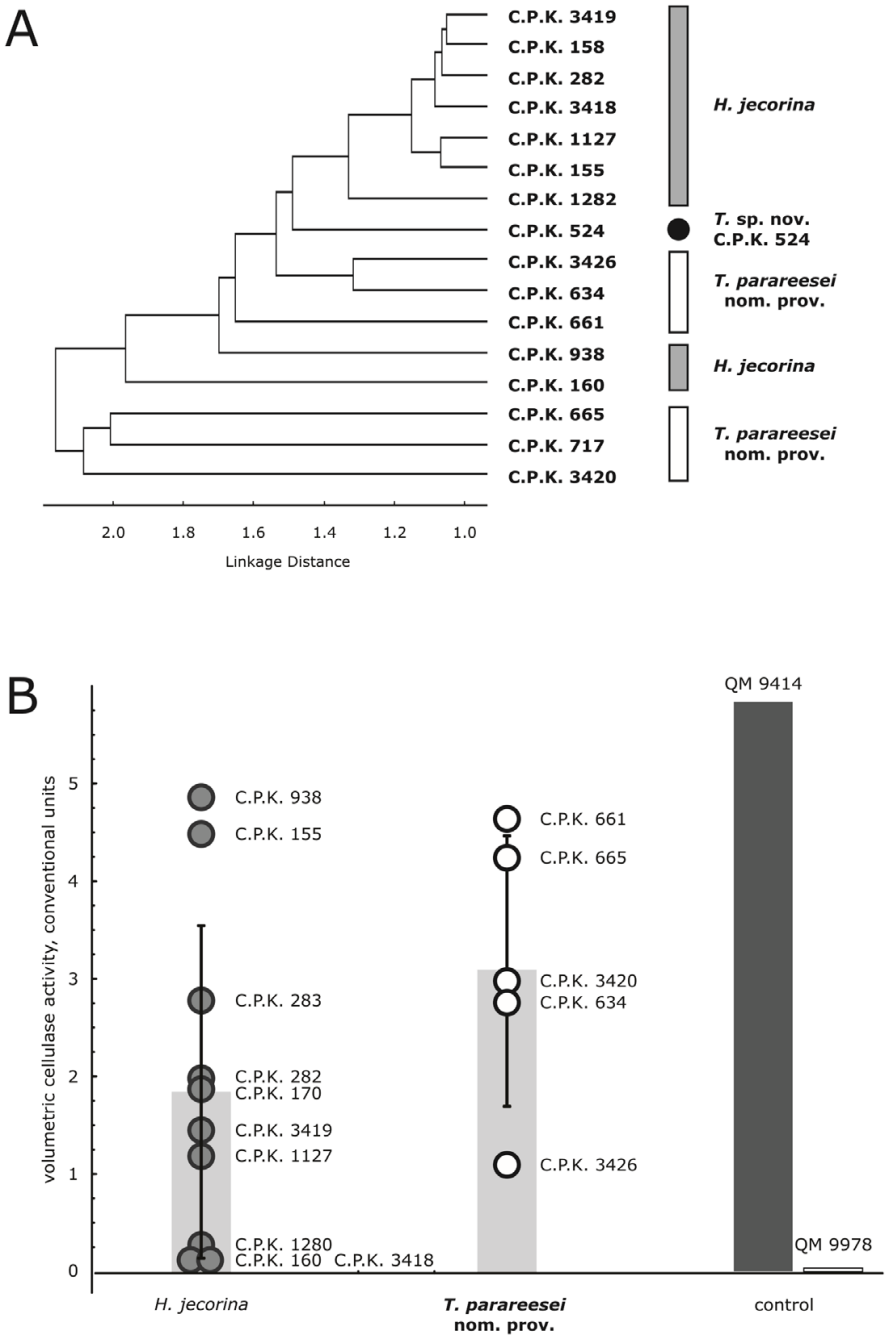
Carbon source utilization by *H. jecorina, T. parareesei* nom. prov. and production of extracellular cellulases. (A) Results of the single linkage cluster analysis (Pearson distance) applied to strains and based on growth on 95 carbon sources and water (Biolog FF MicroPlate ™) inferred from optical density values at 750 nm after 48 hours of incubation (linear growth stage) under ambient illumination conditions. (B) Volumetric cellulase activity of *H. jecorina* and *T. parareesei* nom. prov. Bars correspond to the average values per species and control strains with standard deviations (vertical lines), circles show the values obtained for individual strains. Control corresponds to cellulase overproducing and cellulase negative mutant strains QM 9414 and QM 9978 respectively, both derived from *T. reesei* QM 6a.

With respect to carbon metabolism, *H. jecorina* and the two new species exhibited qualitatively very similar carbon source utilization profiles. The full scale profile will be published elsewhere alone with the formal description of the new species; the list of carbon sources is in Table S1 and in Druzhinina et al. [31]. On a quantitative basis, *T. parareesei* nom. prov. and *T.* sp. C.P.K. 524 generally exhibited faster growth rates on so-called “Cluster I” carbon sources, i.e. the ones which provide fastest growth (such the chitin monomer n-acetyl-β-d-glucosamine or the hemicellulose monomers l-arabinose, d-xylose, d-galactose and corresponding polyols; cf. [31]), and also displayed a broader intraspecific variation (Fig. 5A). In contrast, the majority of *H. jecorina* strains showed a much more conserved quantitative pattern of growth on these carbon sources, yet with a significantly lower growth rate (except C.P.K. 938 and C.P.K. 160). Thus, we conclude that *T. parareesei* nom. prov. has become more versatile and efficient in the utilization of its preferred carbon sources.

Consistent results were obtained when the experiment was repeated with carbon sources typical for soil (EcoPlate™; Biolog Inc., Hayward, CA, USA, see Table S1 for individual carbon sources). Although the growth of both species on EcoPlates was slower than in the experiment above, the results confirm that in general *T. parareesei* nom. prov. is more competent in utilizing these carbon sources (ANOVA, F(1, 133) = 38.32, *P* = 0.000).

Since the only known anamorphic strain of *H. jecorina* - *T. reesei* QM 6a is a model fungus for cellulase formation, we tested whether the superiority of *T. parareesei* nom. prov. in carbon assimilation would also be reflected in this trait. We therefore tested 10 randomly chosen strains of *H. jecorina* and five strains of *T. parareesei* nom. prov., and included the cellulase twofold-overproducing and the cellulase negative mutants QM 9414 and QM 9978 respectively (Fig. 5B). The results show that both species contain extremely efficient cellulase producing strains which gave values close to that of QM 9414. For *H. jecorina* these were two strains isolated from Brazil (C.P.K. 938 and C.P.K. 155); for *T. parareesei* nom. prov. these are also South American C.P.K. 661 and C.P.K. 665 isolated from northern Argentina. Interestingly, three strains of *H. jecorina* showed no cellulase activity, while all strains of *T. parareesei* nom. prov. were efficient producers. Analysis of variance, however, did not detect any statistically significant difference between two species (ANOVA, *P*>0.05). This suggests that the ability to produce cellulases for the degradation of cellulose in the environment has been maintained in both species.

#### Photosensitivity

Light sensing is an important mechanism in the ecophysiological adaptation of fungi as it is strongly involved in regulation of their reproduction. Seidl et al. [6] reported that light is important for formation of perithecia of H. jecorina. Friedl et al. [32] demonstrated that light plays the role in the conidiation of H. atroviridis. Our recent studies have shown that light influences the mycelial growth of Hypocrea but the effect varies depending on the species: temperate H. atroviridis is strongly stimulated by illumination [33] while mutant strains derived from tropical T. reesei are frequently photoinhibited [34]. Thus, photosensitivity may reflect the ecological niche of the fungus.

**Figure 6:**
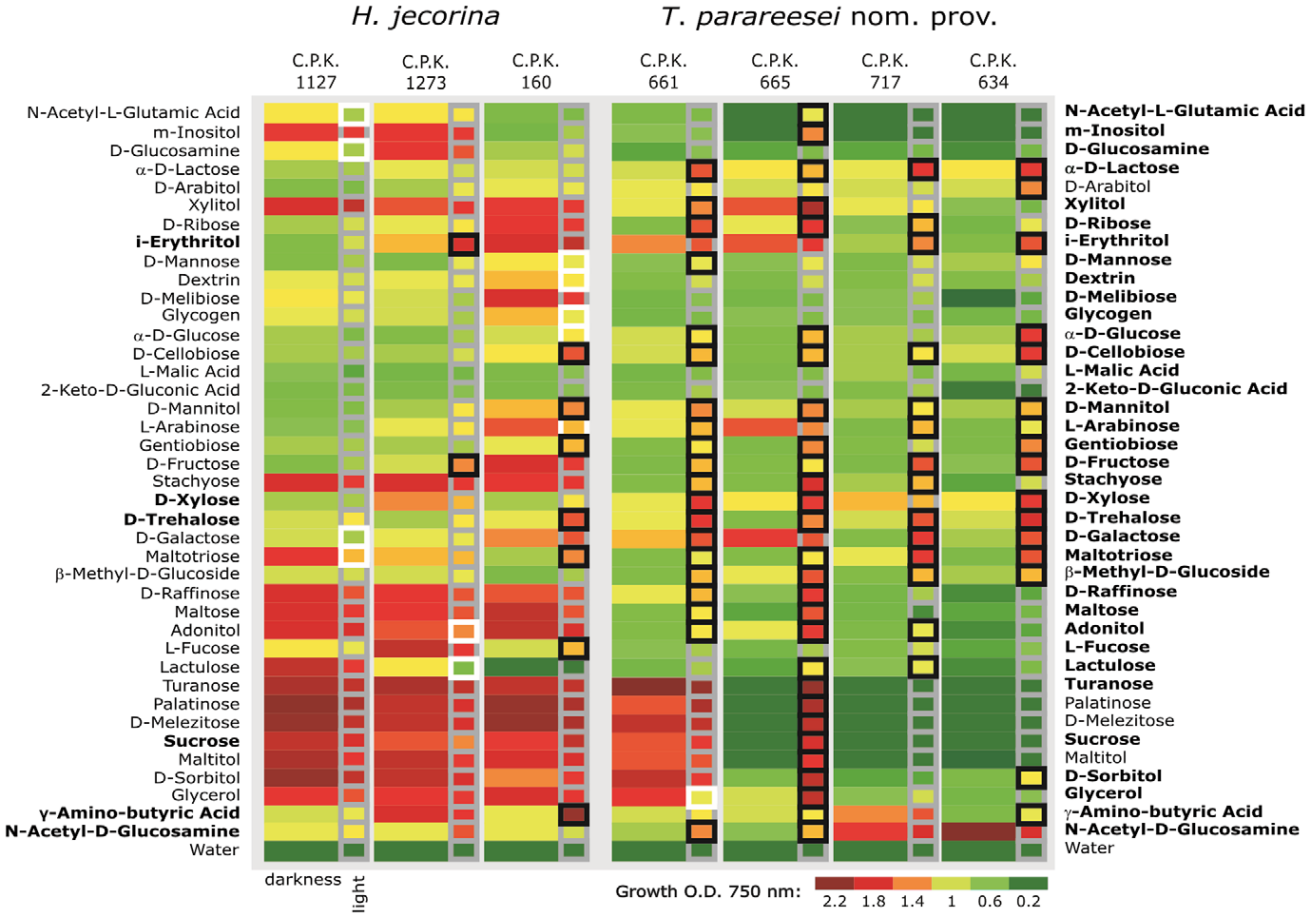
Photosensitivity map of *H. jecorina* and *T. parareesei* nom. prov. Photosensitivity map of *H. jecorina* and *T. parareesei* nom. prov. constructed based on the two way joining cluster analysis. Framed squares show growth under conditions of sun light: white, black and grey frames correspond to photoinhibition, photostimulation and neutral photoresponse respectively. Bold font used for carbon sources indicates those which supported conidiation of *H. jecorina* (left list) and *T. parareesei* nom. prov. (right list) respectively.

Because of the differences in carbon assimilation, we reasoned that the response to light may also be different in *H. jecorina* and *T. parareesei* nom. prov. We thus incubated Biolog FF MicroPlates either in complete darkness or under the conditions of day light (May-June, N 48°) for 72 hours and compared the data (Fig. 6). Indeed, all strains of *H. jecorina* show their best growth rates in darkness, which is basically not changed by exposure to light. We have detected about 10 cases of photoinhibition (on individual carbon sources, see Fig. 6), which were strain- but not species-specific. Growth of only one strain, C.P.K. 160, was considerably stimulated by light on several carbon sources (for example, d-cellobiose, d-mannitol, gentiobiose, d-trehalose, maltotriose and l-fucose). In contrast, the best growth of the majority of *T. parareesei* nom. prov. (all possessing the *MAT1-1*) was under illumination indicating that this species is not capable of normal growth in darkness (Fig. 6). No cases of photoinhibition were detected for *T. parareesei* nom. prov. These data show a striking difference in physiological adaptation of two species and confirm that although they are sympatric they have different ecological niches. The detection of photostimulation in *T. parareesei* nom. prov. is particularly interesting as all its strains were isolated from soil.

#### Conidiation

*H. jecorina* has a complete holomorphic life cycle and forms both conidia and ascospores, while propagation of *T. parareesei* nom. prov. is dependent on the distribution of its mitospores. A visual inspection of cultures indicated that *T. parareesei* nom. prov. conidiates essentially more intensively than the mycelial forms of *H. jecorina* which leads to the striking difference in culture morphology (data not shown). Since conidiation is carbon source dependent [33], we tested it on 95 carbon sources. The results show that in total *H. jecorina* conidiates only on 7 out of 95 carbon sources (6 of those shown on Fig. 6, see the corresponding legend) while *T. parareesei* nom. prov. forms mitospores on 62 carbon sources (36 of those shown on Fig. 6). A quantification of conidial density per cm^2^ of a MEA plate showed that on an average *H. jecorina* formed 3.6 (±1.78)×10^6^ per 10 tested strains, while the six available strains of *T. parareesei* nom. prov. produced an average of 16.05 (±4.8)×10^6^ conidia under the same conditions.

#### Antagonistic potential against soil competent and epigeal plant pathogens

**Figure 7:**
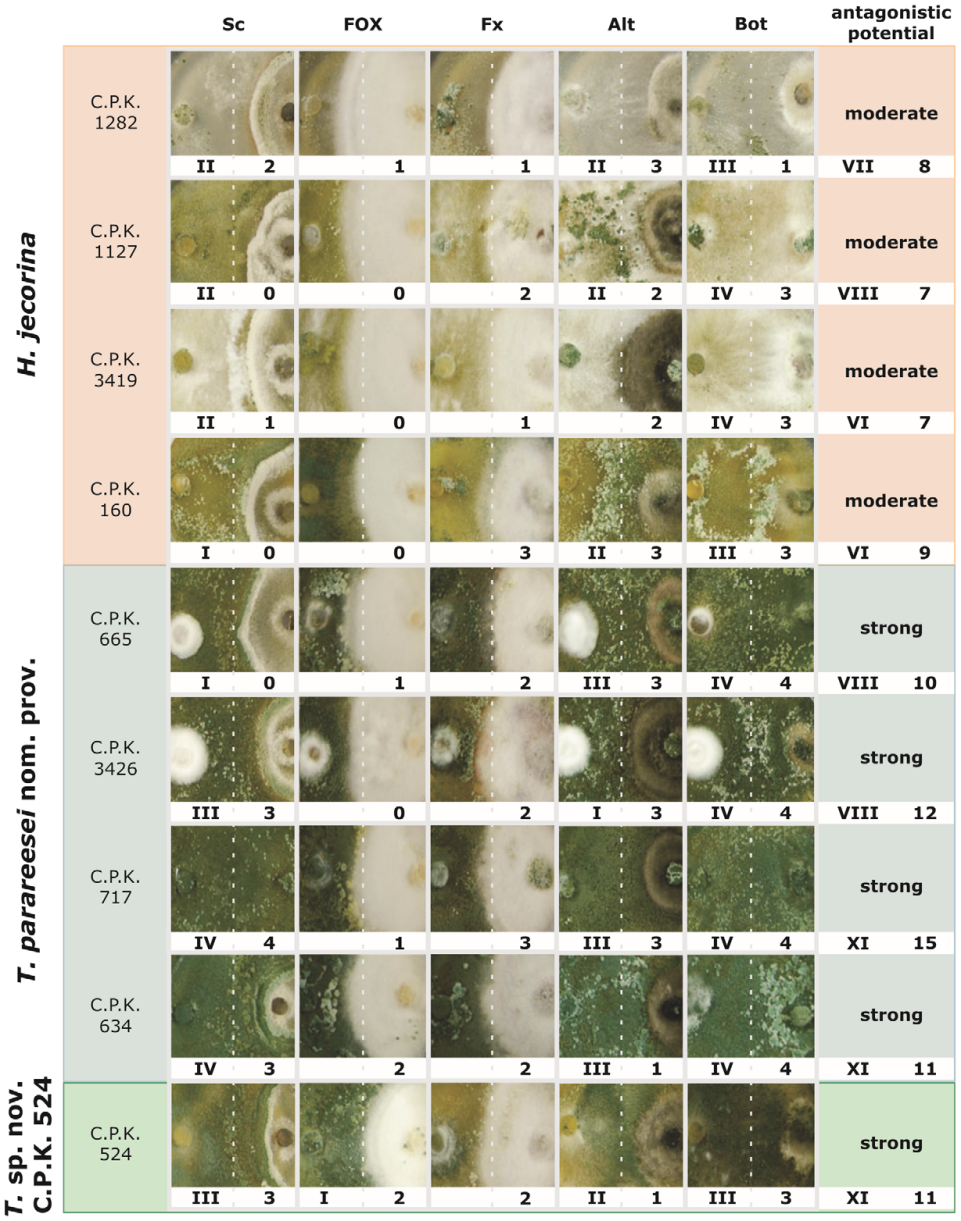
Mycoparasitic ability of *H. jecorina* and *T. parareesei* nom. prov. Results of dual confrontation tests between *Trichoderma* strains (inoculated on the left side) and the plant pathogenic fungi (inoculated on the right side): Sc–*Sclerotinia sclerotiorum*, FOX–*Fusarium oxysporum* complex, Fx–*F. xylarioides*, Alt–*Alternaria alternata*, Bot–*Botrytis cinerea*. Roman numbers indicate the weak (I), moderate (II), strong (III) and very strong (IV) ability of *Trichoderma* to inhibit the growth of the prey fungus. The ability to overgrow the mycelium of prey fungi is given in Arabic numbers on the similar scale. Antagonistic potential is calculated as the mean value for a strain to combat all five pathogens. The dashed lines correspond to the center position between confronted fungi.

Species of *Hypocrea/Trichoderma* are renowned for their mycoparasitic behaviour [1]. In order to test whether the two species may also differ in their antagonistic abilities, we have selected five plant pathogenic fungi which differ in their primary habitat: *Sclerotinia sclerotiorum* and *Fusarium oxysporum* (FOX, *F. oxysporum* species complex) representing soil competent mycobionts; and *F. xylarioides*, *Alternaria alternata* and *Botrytis cinerea* representing pathogens of green plant tissue and thus predominantly epigeal fungi. Fig. 7 shows the summary of dual confrontation tests assessed after 10 days of co-cultivation on MEA medium. *H. jecorina* is able to considerably inhibit growth of *S. sclerotiorum, A. alternata* and *B. cinerea*. The latter plant pathogen was so completely combated by *H. jecorina* C.P.K. 160 and C.P.K. 1127 that its mycelium was overgrown and killed. No antagonism to both *Fusarium* species by *H. jecorina* was detected. Strains of *T. parareesei* nom. prov. showed a conserved pattern of mycoparasitic activity with almost no variation among strains, and they also exhibited a superior antagonistic potential against *S. sclerotiorum*, *B. cinerea* and particularly *A. alternata*. *T*. sp. C.P.K. 524 behaved similar to *T. parareesei* nom. prov. except that it was the most resistant strain against the isolate of FOX used. We should like to stress that the *in vitro* dual confrontation tests are only an indirect means to assess antagonistic potential of the fungus. However, the significant difference shown between *H. jecorina* and *T. parareesei* nom. prov. suggests that the former is only a moderate mycoparasite with significant variation among isolates, whereas the latter species may exhibit a stronger antagonistic potential against all epigeal fungi tested (Fig. 7).

## Discussion

*T. reesei* QM 6a has been a taxonomic riddle. Originally isolated in 1942, it was a victim of then almost undeveloped taxonomy for *Trichoderma*. Therefore it was first determined to be *T. viride* (because the genus was in that time believed to consist only of this single species [35]), and later on recognized as a unique species and named in honor of its detector Elwyn T. Reese *T. reesei* [36]. Bissett [37] then revised it as being co-specific with *T. longibrachiatum*. Finally, it was recognized to be identical to the pantropical ascomycete *H. jecorina* [6], which was itself just distinguished as a separate tropical species closely related to *H. schweinitzii* [22]. Yet small morphological differences and the inability of these authors to cross it with other *H. jecorina* isolates in the lab led Kuhls et al. [6] to assume that it is actually a clonally derived asexual form of *H. jecorina*.

The present data have clearly rejected this hypothesis: our analysis shows that the original isolate *T. reesei* QM 6a reveals a history of recombination similar to that of the teleomorphic isolates of *H. jecorina*. These findings are also supported by our recent discovery that *T. reesei* QM6a is a *MAT1-2* idiotype and can indeed be crossed with *MAT1-1* partners of *H. jecorina* [8]. Previous failures to obtain crossing is therefore likely due to the use of the incorrect mating type partner or inappropriate experimental conditions. *T. reesei* clearly is an isolate identical to *H. jecorina*, and the minor differences in anamorph morphology [6] and nutrient assimilation [38] to other (more recent) isolates of *H. jecorina* are likely the result of its maintenance in the laboratory for the last 65 years.

A large number of studies have shown that fungal taxa which were defined on the basis of consistent invariant morphological features in fact contain multiple, well differentiated phylogenetic species [9], [12]–[15]. Here we provide a further example for this growing list, the pantropical ascomycete H. jecorina, and show that the 33 strains that were available for this study actually consist of not less than four different species, i.e. H. jecorina, T. parareesei nom. prov., T. sp. nov. C.P.K. 524 and H. sp. nov. G.J.S. 85–238. The formal taxonomic description of T. parareesei nom. prov. will be published elsewhere (L. Atanasova, W.J. Jaklitsch, C.P. Kubicek, and I.S. Druzhinina, manuscript in preparation) but we will refrain from describing the other two phylogenetic taxa based on the small number of strains. Taxonomies with arbitrarily named fungal species (T. parareesei nom. prov. in the present case) have frequently been published [11]–[14] and enable researchers to communicate effectively before the formal species nomenclature has been established [9].

*H. jecorina* yielded evidence for sexual recombination even via a large distance and geographic barriers, thus indicating the presence of a very efficient system for transfer of spores, and/or of the fungus itself (e.g. via wood logs, insects etc.). On the other hand, the same tests clearly rejected this possibility for *T. parareesei* nom. prov. Thus, these two species represent a diverged species pair, similar to *H. orientalis* and *T. longibrachiatum* [15].

Our findings show that those strains of *H. jecorina*, which were recently isolated as anamorphs from soil and litter and identified as *H. jecorina* by morphological analysis and molecular barcodes [17] are in fact a cryptic agamospecies, *T. parareesei* nom. prov. It is very intriguing in this regard that this sibling anamorphic sister species of *H. jecorina* is similarly restricted to the same narrow belt of±20° altitude around the equator, and otherwise similarly pantropical. It consequently represents a rare example of sympatric speciation (i.e. the evolution of reproductive isolation between co-distributed populations) of a saprotrophic fungus [39].

One of the most obvious reasons for such a divergent speciation resulting in formation of an agamospecies would be a mutation in a gene required for sexual reproduction. In such a scenario *T. parareesei* nom. prov. would have arisen from its ancestor because of a loss of a subpopulation ability to mate. However, our data reject this hypothesis as in such a case all strains of the anamorphic population should have the same mating type. The possession of both *MAT1-1* and *MAT1-2* loci, although unevenly distributed in our (small) sample, suggest the operation of other speciation mechanisms. The other indirect argument against altered mating mechanism as the driving force for speciation comes from the analysis of topologies of phylogenetic trees. If *T. parareesei* nom. prov. would have arisen as an agamospecies by a sudden loss of its ability to sexually recombine, its gene sequences should display lower evolutionary rates compared to *H. jecorina*. In other words the length of the branch leading to *T. parareesei* nom. prov. from the hypothetical ancestor of both species should be then shorter than the one leading to *H. jecorina*. However, the individual trees presented here as well as the analysis of an exon of the gene coding for RNA polymerase subunit B II (C.P. Kubicek, I.S. Druzhinina, unpublished data) revealed similar genetic distances between *H. jecorina* and *T. parareesei* nom. prov. from their hypothetical ancestor respectively.

The currently most favored explanation for reproductive isolation postulates that hybrid inferiority is caused by antagonistic epistasis between incompatible alleles at interacting loci [40]–[42]. Theoretical models have shown that sympatric speciation may occur when the same genes control both mating and habitat preference or fitness [43]. Our data would be compatible with this hypothesis: growth and conidiation of *MAT1-1* strains of *T. parareesei* nom. prov.–in contrast to *H. jecorina* - are stimulated by intensive illumination, whereas the *MAT1-2* strain behaves similarly to *H. jecorina*. Evidence for regulation of both mating and carbon source utilization by the blue light has previously been obtained [32]–[34] demonstrating that sexual reproduction and carbon assimilation may indeed share the same regulatory circuits. The availability of further *MAT1-2* strains of *T. parareesei* nom. prov. will help to differentiate between the effect of mating type loci and ecophysiological divergence of two species.

Sympatric speciation is well known from the evolution of plant and human pathogenic fungi [39], but has not been reported for non-pathogenic saprotrophs like *Hypocrea/Trichoderma*. In this work we applied a bioinformatic sequence analyses and ecophysiological characterization to obtain some insights into the forces thriving speciation in *H. jecorina* and *T. parareesei* nom. prov. We have analyzed the global versatility of their carbon metabolism, response to light, conidiation intensity and mycoparasitic potential (for a summary see Table 3). The differences detected are indeed striking and reveal that *T. parareesei* nom. prov. and *H. jecorina* occupy different ecological niches in the vertical profile of the tropical forest. *T. parareesei* nom. prov. displays all the properties of an environmental opportunist: it shows faster growth on a wider spectrum of carbon sources than *H. jecorina*, and produces a much higher number of propagules on a greater variety of carbon sources. The species is also able to strongly compete with other mycobionts and is mycoparasitic on the epigeal plant pathogenic fungi tested. The latter fact, combined with its profound photostimulation of conidiation, allows us to speculate that *T. parareesei* nom. prov. might occupy an ecological niche connected with photosynthesizing parts of higher plants, i.e. the canopy of the tropical forest.

**Table 3:** Comparative ecophysiology of *H. jecorina* and *T. parareesei* nom. prov.

	*H. jecorina*	*T*. *parareesei* nom. prov.	Reference
**Known substrata**			
teleomorph	wood deris	n. a.	Table 1
anamorph	*unknown*	Soil	Table 1
**Phenetic profile**			
growth rate	moderate	Fast	
carbon metabolism	invariable	variable	Fig. 5A
cellulase secretion	variable to high	high	Fig. 5B
soil competence	low	moderate	
extracellular yellow pigmentation	strong	weak	Fig. 7
photosensitivity reaction	insensitive to inhibited	stimulated	Fig. 6
photoadaptation to	darkness	illumination	
**Conidiation**			
density (per cm^2^)	(36±17.8)×10^5^	(160.5±47.7)×10^5^	n. a.
carbon sources supporting conidiation (out of 95)	7	62	Fig. 6
formation rate	low	High	
**Antagonistic potential**			
against soil fungi	low to moderate	moderate to strong	Fig. 6
against epigeal fungi	moderate	strong	

n.a. corresponds to not applicable

*H. jecorina* in turn seems to be a specialized on a narrow habitat, where its surviving strategy mainly relies on the advantages of sexual reproduction. The species has definitely reduced conidiation efficiency (compared for the general mean for the genus, I.S. Druzhinina, personal observations) and is less aggressive against potentially competing fungi. It is remarkable that the quantitative pattern of carbon metabolism of *H. jecorina* is highly conserved (with exception of two fast growing strains, Fig. 5A) which may indicate its nutritional specialization. The fact that its anamorph has only very rarely been found in its natural environment [38] further supports the hypothesis that this species is strongly specialized. In our own work on the assessment of the general *Hypocrea/Trichoderma* biodiversity, we failed to encounter *H. jecorina* in an asexual form in more than 1000 samples, collected worldwide from soil and litter. It is probable that asexual reproduction in *H. jecorina* - like it apparently occurred in the case of the original strain of *T. reesei* and the three strains described in [38] - can be observed only under certain conditions or in certain habitats. Yet the origin of the anamorphic strains G.J.S. 97–177 (CBS 102271) and G.J.S. 97–178 (CBS 102270) both found on dead cacao brooms in Brazil, and G.J.S. 97–38 (CBS 999.97), from soil at a storage lake in French Guiana [38] does not provide a hint towards ecological adaptation of the species. Moreover details of the environment at the US Army camp in Guadalcanal during WW II, where and when *T. reesei* QM 6a was originally isolated, are also not available. Further samplings in tropical regions may eventually disclose the habitat of one of the most prominent producer in biotechnology.

## Methods

### Material Studied

The strains, their origin and the sequence accession numbers used in this work are listed in Table 1. The isolates are stored at −80°C in 50% glycerol in the laboratory of Vienna University of Technology (TUW). Strains are grouped according to their identification in the present work. For convenience, TUW-lab codes (C.P.K.) are used for the strains throughout, but other collection numbers are also listed in Table 1.

### Molecular Genetic Analysis

#### DNA extraction, PCR amplification and sequencing

Mycelia were harvested after 2–4 days of growth on MEA at 25°C and genomic DNA was isolated using QIAGEN DNeasy® Plant Mini Kit following the manufacturer's protocol. Amplification of nuclear rRNA gene cluster, containing the ITS1 and 2 and the 5.8S rRNA gene, and of fragments of *tef1* (translation elongation factor 1-α) and *cal1* (calmodulin) was performed as described previously [15]. The *las1* gene (fgenesh5_pg.C_scaffold_1000016), which encodes the orthologue of an essential nuclear protein regulating bud formation and morphogenesis in *S. cerevisiae* [21] was amplified using primers given in Table 4. PCR amplification was carried out in an *i*-cycler (BIO-RAD, USA) for 30 cycles of 94°C for 1 min denaturing, 58°C for 1 min annealing, and 74°C for 50 sec extension. Initial denaturing was at 94°C for 1 min.and the final extension was at 74°C for 7 min. PCR amplification of the mating type loci and the mating type genes was carried out as described [8]. The primers used for *MAT1* loci are listed in Table 4. PCR fragments were purified (PCR purification kit, Qiagen, Hilden, Germany), and sequenced at MWG (Ebersberg, Germany).

**Table 4:** Selected PCR primers used in this study.

Gene or locus	Primer pair	Sequence (5′-3′)
*Las1*	LAS1 fw	CATCGACGTATGCTGTGAGG
	LAS1 rev	CTTGGCGATGGAGTACATGC
*MAT1-1* and *MAT1-2*	aF	CATCGAAGCATCTACCTACTTG
	aR	CGAAGCGAAACACACGAC
*mat1-1-1*	m1-1F	TCCTCTCAATGCGTTCATGGC
	m1-1R	AGAAGATCATTCTCTGTGTTGGGA
*mat1-1-2*	m2-1F	CTCGAGAGGGATATACACCAG
	m2-1R	CTTCCTACACGGATGCCAGA
*mat1-1-3*	m1-3F	ATCTCCATGTCTTTATTCCCGAAG
	m1-3R	CTTGTAGTCGGGATATTTCTCC
*mat1-2-1*	m2-1F	GCGCACCACGGTATTTCATTG
	m2-1R	ATTTGCGCGGCTTGTATTGG

#### Phylogenetic analysis

For the main phylogenetic analysis DNA sequences were aligned with Clustal X 1.81 [44] and then edited using GeneDoc 2.6 [45]. The possibility of intragenic recombination, which would prohibit the use of the respective loci for phylogenetic analysis, was tested by linkage disequilibrium based statistics as implemented in DnaSP 4.50.3 [46]. The neutral evolution of coding fragments (*cal1* and *las1*) was tested by Tajima's test implemented in the same software. The interleaved NEXUS file was formatted using PAUP*4.0b10 [47]. The best nucleotide substitution model for the each locus was determined using jMODELTEST [48]. As Akaike and Bayesian Information criteria (AIC [49] and BIC [50] respectively) selected different nucleotide substitution models for every locus and due to the relatively small size of individual datasets (1242 characters per 34 sequences for the biggest) the unconstrained GTR + I + G substitution model was applied to all sequence fragments (Table 2). Metropolis-coupled Markov chain Monte Carlo (MCMC) sampling was performed using MrBayes v. 3.0B4 with two simultaneous runs of four incrementally heated chains that performed 5 million generations. The length of run (number of generations) for each dataset was determined using AWTY graphical system [51] to check the convergence of MCMC. Bayesian posterior probabilities (PP) were obtained from the 50% majority rule consensus of trees sampled every 100 generations after removing the first trees using the “burnin” command. Number of discarded generations was determined for each run based on visual analysis of the plot showing generation versus the log probability of observing the data. PP values lower than 0.95 were not considered significant while values below 0.9 are not shown on the resulting phylograms. Model parameters summaries after MCMC run and burning first samplings as well as nucleotide characteristics of used loci are collected in Table 2.

#### Detection of recombination

he congruence or incongruence of the three gene genealogies was used to infer recombination between isolates. To this end, three different tests were employed: the incongruence length difference/partition homogeneity test (ILD/PHT) [27], [28] using a score of *P*<0.05 to reject the null hypothesis of congruence between loci; the Index of Association (IA) test [29], in which the data were compared to the IAs of artificially recombined datasets; and the Phi-test implemented in SplitsTree [26], which uses the pairwise homoplasy index, PHI ( =  Φ) statistic, to detect refined incompatibility indicating recombination [30].

In addition we applied split decomposition implemented in the SplitsTree program, version 4.0 [25], [26], using pairwise distances under the Kimura 3ST model [52].

#### Mating type RFLP analysis

The ca. 10 kb large PCR fragments of the complete *MAT*-loci and their flanking regions were digested with *Pst*I (Fermentas, Burlington, Canada). Sequences of *H. jecorina* MAT1-1 and MAT1-2, derived from strain C.P.K. 2189 (CBS 999.97, [6], [8], [38]) were used as reference strains in the mating experiments.

### Ecophysiological Characterization

#### Phenotype profiling

The carbon assimilation patterns were investigated using Biolog FF MicroPlate™ and EcoPlate™ (Biolog Inc., Hayward, CA, USA) according to the protocol published recently [31]. The complete lists of carbon sources implemented in both plates are given in Table S1. Briefly, strains were grown on 3% malt extract agar (MEA), and 90 µl of a conidial suspension from them (75±2% transmission at 590 nm) was dispensed into each of the wells of a corresponding Biolog microplate. Inoculated microplates were incubated at 28°C, and optical density (O.D.) determined after 12, 18, 24, 36, 42, 48, 66 and 72 h at 750 nm. Analyses were repeated at least three times for each strain.

In order to estimate the effect of illumination on mycelial growth and conidiation the protocols of Friedl et al. [32], [33] were used respectively. Biolog FF MicroPlates were incubated either at natural day light (May-June, N 48°), while plates for darkness experiment were not exposed to any light source in between the measurements. Biolog EcoPlates were incubated in darkness.

Data exploratory statistical analyses were performed using Statistica 6.1 (StatSoft, Inc., Tulsa, OK, USA) data analysis software system.

#### Antagonistic potential

To assess the antagonistic potential of anamorphic cultures of *H. jecorina* five potential prey fungi have been selected: *Sclerotinia sclerotiorum* C.P.K. 3593 and FOX (*Fusarium oxysporum* species complex, strain C.P.K. 1842) to represent soil and rhizosphere competent pathogens and *F. xylarioides* C.P.K. 3453, *Alternaria alternata* C.P.K. 3594 and *Botrytis cinerea* C.P.K. 3592 to represent epigeal plant pathogens. Potential prey fungi were inoculated as agar blocks of the standard size always 1 cm from the edge of the Petri plate and pre-cultivated on 3% PDA in darkness at 25 C. Then similar agar blocks with *Trichoderma* cultures were introduced on the opposite side of the plate and cultivated for 10 days. Antagonistic potential was semi-quantified based on both ability to inhibit the growth of a pathogen and ability to overgrow the mycelium of the pathogenic fungus. One of 5 phases for each confrontation was recorded: 0–no inhibition; I - started to inhibit; II - clear signs of inhibition; III - mostly or strongly inhibited; IV - totally inhibited. The ability to overgrow was based on the same scale but recorded using Arabic numbers.

#### Quantitative and qualitative conidiation assessments

Quantitative assessment of conidiation was done by measuring conidia density per cm2 produced on Petri dishes on 3% MEA after 10 days of cultivation under natural light/darkness cycle. For this purpose the 6.2 cm2 fragment of an agar plate was cut and rinsed in 15 ml of water containing 0.1% of Tween-80 until visually all conidia were washed out. The concentration of conidia was estimated based on optical density at 540 nm (Biolog Turbidimeter) and transferred into density values based on the calibration curve inferred from the serial dilutions of the standard suspension. In addition the qualitative conidiation assessment when the ability to produce conidia was estimated in respect to carbon metabolism was done using Biolog FF Microplates. In this case conidiation intensity was estimated according to the nominative scale [32] after 72 and 168 hours of incubation. Values 0 and 1 were assigned to the cases when no aerial mycelium and no conidia were detected respectively. Values above 1 corresponded to different intensities of conidiation from single spores (2) to the full coverage of the microplate well by a thick conidial mat (4).

#### Cellulase production

Strains were grown in 1-liter Erlenmeyer flasks on a rotary shaker (250 rpm) at 28°C for 72 h in 250 ml of Mandels-Andreotti medium [53] containing 1% (wt/vol) Avicel microcrystalline cellulose as the sole carbon source. Conidia (final concentration, 10^8^ per liter) were used as the inoculum. Cellulase activity in the extracellular culture supernatant was measured using 4-nitrophenyl-β-d-lactopyranoside as a substrate dissolved in a 50 mM sodium citrate buffer, pH 5.0. Other conditions for the assay were the same as used for β-glucosidase [54]. One unit (1 U) of enzyme activity is given as the amount of enzyme needed to liberate 1 µmole 4-nitrophenole from the substrate per min. under the conditions of the assay.

### Mating Experiments

This was done as described by Seidl et al. [8]. Briefly, the two putative mating partners were placed onto 3% MEA plates 5 cm apart from each other, and incubated at 25°C for 7–10 days in the presence of a natural illumination cycle. In compatibility reactions fruiting bodies were formed at the interaction zone between the two cultures.

## Supporting Information

Figure S1 Single loci phylograms. Bayesian circular phylogram inferred from the concatenated dataset of las1 (A), tef1 (B), cal1 (C) phylogenetic markers. Symbols at nodes correspond to posterior probabilities (PP) >95%.
(1.34 MB TIF)

Figure S2 Amino acid polymorphism of MAT1 sequences. The aa alignments of MAT1-1-2, Mat1-1-3 and MAT1-2 proteins for *H. jecorina*, *T. parareesei* nom. prov. and T. sp. nov. C.P.K. 524 respectively. Arrows indicate polymorphic sites.
(0.39 MB TIF)

Table S1 Carbon sources of BIOLOG FF microplates.
(0.10 MB DOC)
