# Supplementary figures and images for: Evolution and Ecophysiology of the Industrial Producer Hypocrea jecorina (Anamorph Trichoderma reesei) and a New Sympatric Agamospecies Related to It
Source: PLoS One. 2010 Feb 12;5(2):e9191. doi: 10.1371/journal.pone.0009191 (PMC2820547; doi:10.1371/journal.pone.0009191)

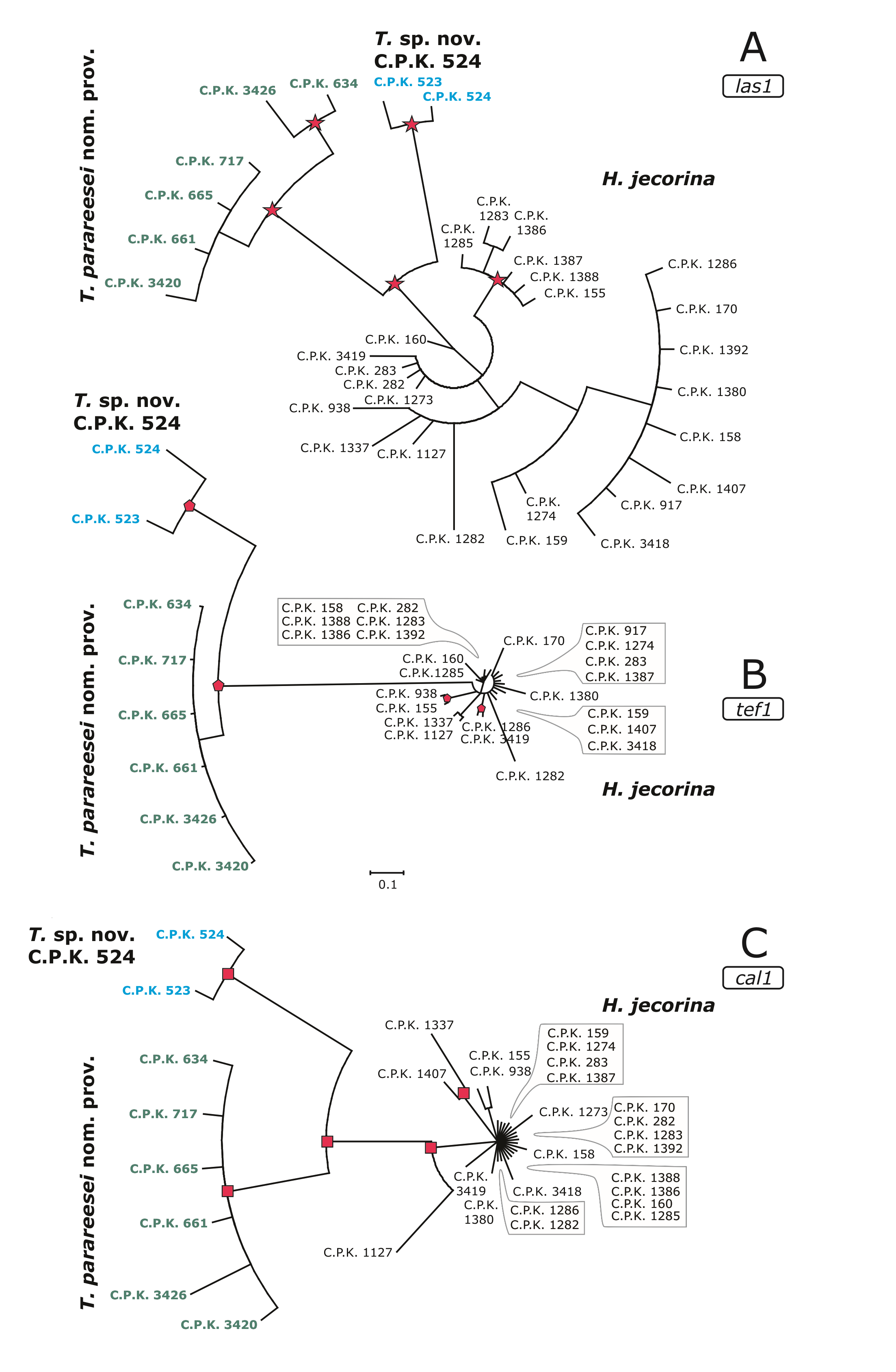

Supplement: Figure S1 — Single loci phylograms. Bayesian circular phylogram inferred from the concatenated dataset of las1 (A), tef1 (B), cal1 (C) phylogenetic markers. Symbols at nodes correspond to posterior probabilities (PP) >95%. (1.34 MB TIF) [file pone.0009191.s001.tif]

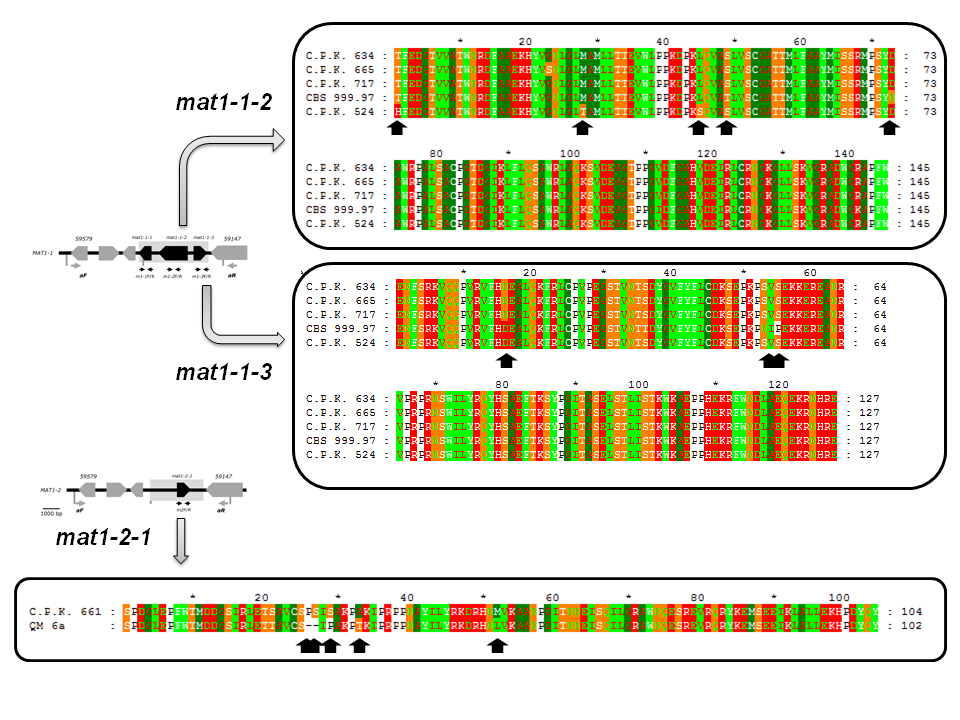

Supplement: Figure S2 — Amino acid polymorphism of MAT1 sequences. The aa alignments of MAT1-1-2, Mat1-1-3 and MAT1-2 proteins for H. jecorina, T. parareesei nom. prov. and T. sp. nov. C.P.K. 524 respectively. Arrows indicate polymorphic sites. (0.39 MB TIF) [file pone.0009191.s002.tif]
