# Supplementary material for: Evolution and Ecophysiology of the Industrial Producer Hypocrea jecorina (Anamorph Trichoderma reesei) and a New Sympatric Agamospecies Related to It
Source: PLoS One. 2010 Feb 12;5(2):e9191. doi: 10.1371/journal.pone.0009191 (PMC2820547; doi:10.1371/journal.pone.0009191)
Supplement: Table S1 — Carbon sources of BIOLOG FF microplates. (0.10 MB DOC) [file pone.0009191.s003.doc]

| BIOLOG plates | Carbon sources |
| --- | --- |
| EcoPlate | β-Methyl-D Glucoside; D-galactonic Acid γ-Lactone; L-arginine; Pyruvic Acid Methyl Ester; D-xylose; D-Galacturonic Acid; L-Aspargine; Tween 40; i-Erythritol; 2-Hydroxy Benzoic Acid; L-phenylalanine; Tween 80; D-Mannitol; 4-Hydroxy Benzoic Acid; L-Serine; α-Cyclodextrin; N-Acetyl-D-Glucosamine; γ-Hydroxybutyric Acid; L-Threonine; Glycogen; D-Glucosaminic Acid; Itaconic Acid; Glycyl-L-Glutamic Acid; D-Cellobiose; Glucose-1-Phosphate; α-Ketobutyric Acid; Phenylethyl-amine; α-D-Lactose; D,L-α-Glycerol Phosphate; D-Malic Acid ; Putrescine |
| FF Microplate | γ-amino-butyric acid; D-mannose; N-acetyl-D-glucosamine; i-erythritol; D-fructose; D-trehalose; D-arabitol; D-mannitol; D-galactose; α-D-glucose; dextrin; glycogen; D-cellobiose; L-arabinose; gentobiose; L-pyroglutamic acid; D-xylose; stachyose; glycerol; maltotriose; L-alanine; α-D-lactose; L-alanyl-glycine; β-methyl-D-glucoside; xylitol; arbutin; L-glutamic acid; lactulose; D-ribose; D-sorbitol; salicin; Quinic acid; L-fucose; maltose; amygdalin; L-asparagine; D-melibiose; β-methyl-D-galactoside; α-D-glucose- 1 -phosphate; adenosine; fumaric acid; D-psicose; succinic acid mono-methyl ester; succinic acid; L-ornithine; adonitol; D-raffinose; D-glucosamine; L-malic acid; L-aspartic acid; 2-keto-D-gluconic acid; D-gluconic acid; D-arabinose; α-ketoglutaric acid; L-serine; L-proline; D-saccharic acid; D-glucuronic acid; g-hydroxybutyric acid; L-lactic acid; α-methyl-D-galactoside; bromosuccinic acid; Tween 80; D-galacturonic acid; glycyl-L-glutamic acid; β-cyclodextrin; turanose; L-alaninamide; palatinose; sucrose; sebacic acid; D-tagatose; sedoheptulosan; m-inositol; adenosine-5 '-monophosphate; L-phenylalanine; putrescine; L-rhamnose; D-malic acid; N-acetyl-L-glutamic acid; α-cyclodextrin; succinamic acid; α-methyl-D-glucoside; L-sorbose; r-hydroxyphenylacetic acid; uridine; 2-aminoethanol; maltitol; β-hydroxybutyric acid; L-threonine; D-melezitose; D-lactic acid methyl ester; glucuronamide; N-acetyl-β- D-mannosamine; water; N-acetyl-D-galactosamine |

Supporting information table 1
